# Gut Microbiota Diversity and Composition Across Shift Types and the Effects of Walnut Supplementation—An Observational and Interventional Study

**DOI:** 10.3390/ijerph23020169

**Published:** 2026-01-29

**Authors:** Sophie Bucher Della Torre, Aurélien Clerc, Pascal Wild, Angeline Chatelan, Jacques Schrenzel, Nadia Gaïa, Chiraz Chaabane, Vladimir Lazarevic

**Affiliations:** 1 Department of Nutrition and Dietetics, Geneva School of Health Sciences, HES-SO University of Applied Sciences and Arts Western Switzerland, 1227 Geneva, Switzerland; 2 PW Statistical Consulting, 54520 Laxou, France; 3 Genomic Research Laboratory, Geneva University Hospitals, University of Geneva, 1206 Geneva, Switzerland; 4 Bacteriology Laboratory, Department of Diagnostics, Geneva University Hospitals, 1205 Geneva, Switzerland

**Keywords:** shift workers, gut health, circadian rhythms, nuts, diet

## Abstract

**Highlights:**

**Public Health Relevance—how does this work relate to a public health issue?**

- Shift work is a widespread occupational exposure associated with circadian disruption and increased risk of chronic metabolic diseases.
- Alterations in gut microbiota diversity may represent a biological pathway linking night shift work, diet quality, and long-term health outcomes.

**Public Health Significance—why is this work of significance to public health?**

- Using a within-person design, this study shows that overall gut microbiota composition remains largely stable across shift types, while night shifts may transiently reduce microbial diversity.
- Healthier dietary patterns were consistently associated with greater gut microbiota diversity, and walnut supplementation appeared to attenuate diversity loss during night shifts.

**Public Health Implications—what are the key implications or messages for practitioners, policy makers and/or researchers in public health?**

- Targeted nutritional strategies focusing on overall diet quality may help preserve gut microbiota diversity in shift-working populations.
- These findings support further public health research on dietary and timing-based interventions to mitigate the health effects of circadian disruption in shift workers.

**Abstract:**

Shift workers are at elevated risk of chronic diseases due to circadian rhythm disruption, suboptimal lifestyle behaviors, and potentially altered gut microbiota (GM). This study investigated variations in GM diversity and composition across three weekly shifts in rotating shift workers and following walnut supplementation. Using a within-person design, GM diversity and composition were compared in 13 shift workers during morning, afternoon, and night shifts. After a three-week observational period, participants added a daily serving of walnuts to their habitual diet for an additional three weeks. GM was analyzed via 16S rRNA sequencing, assessing diversity and bacterial composition across shift types and between the observational and interventional phases. Overall GM composition did not differ between the beginning and end of shifts, by shift type, or following walnut supplementation. Bacterial diversity remained stable except for a significant decrease at the end of the night shift during observation (*p* = 0.03), which was not observed during walnut supplementation. GM clustered strongly by subject, and a healthier diet correlated with greater mean GM diversity (r = 0.64, *p* = 0.02). Despite overall GM stability, the decline in diversity during night shifts suggests that targeted nutritional strategies, such as walnut supplementation, may help preserve gut health in shift workers.

## 1. Introduction

Shift workers are a growing population. In Switzerland, about one in five workers has a rotating work schedule [1]. It is well established that shift workers face an increased risk of developing chronic diseases such as obesity, type 2 diabetes, cardiovascular diseases, and some types of cancer [2,3,4]. However, the underlying mechanisms remain unclear, and various factors, such as internal circadian desynchronization, unhealthy lifestyle behaviors, and lack of sleep, interact in complex ways. The human circadian system has developed to anticipate rhythmic alternation between periods of wakefulness and food intake, and periods of rest and fasting [5]. Circadian rhythms influence many physiological processes, including gastrointestinal and metabolic functions [6]. In shift workers, altered eating and sleeping patterns tend to impair synchronization of the central clock located in the hypothalamus and independent peripheral clocks located in all major tissues [7].

Gut microbiota (GM) may also play an important role in the increased risk of chronic diseases in shift workers [8]. GM, defined as the diverse microbial community colonizing the host’s gastrointestinal tract [9], contributes greatly to host physiology by providing metabolic and immunological functions [10]. GM dysbiosis has been associated with metabolic diseases such as obesity, type 2 diabetes, and cardiovascular diseases [11,12,13]. The timing of food intake also seems to influence the GM composition. Indeed, evidence has shown that the GM, like its host, follows a circadian rhythm largely regulated by food timing [14]. These diurnal fluctuations resulted in time of the day-specific taxonomic configurations and functional capacities, with substantial effects on host immunity and metabolism [15,16]. Diurnal oscillations of the GM can influence host circadian rhythms [17], but they also depend on the functional circadian clock of the host [16]. Consequently, the disruption of normal sleep patterns, such as shift work or chronic jet lag, impairs diurnal rhythmicity in the GM [17]. In addition, circadian disruption associated with shift work affects the secretion of key hormones, including cortisol, ghrelin, and leptin, which regulate stress responses and energy balance [18]. These hormonal changes may contribute to GM alterations via the gut–brain axis. In particular, cortisol directly has been shown to influence gut function, including gut permeability [19].

GM composition is characterized by substantial interindividual variability, while remaining relatively stable within individuals over time [20]. Long-term dietary patterns are strongly associated with distinct GM profiles [21]. However, some intervention studies have shown GM changes in response to drastic dietary shifts, such as switching from an animal-based diet to a plant-based diet [22] or significantly altering the macronutrient composition of the diet [23]. Several interventional studies have also highlighted the prebiotic properties of several types of nuts and their potential to improve GM composition and function [24]. Indeed, fermentation of dietary fiber and polymerized polyphenols found in nuts by GM forms putatively beneficial bioactive molecules, such as butyrate, which have been associated with the transition to a healthier GM [25].

This evidence paves the way for innovative interventions using food-based prebiotics as a potential approach to alleviate circadian rhythm misalignment and related metabolic diseases, ultimately helping to prevent chronic disease development in shift workers [17]. However, several research gaps remain, such as clarifying the degree of dysbiosis caused by shift work alone and understanding the interactions between diet and GM. Therefore, the present study aimed to investigate whether and how the diversity and composition of shift workers’ GM vary across different shifts and whether walnut supplementation influences these patterns.

## 2. Materials and Methods

This study consisted of two periods. In the first 3-week observational period, we compared GM diversity and composition of shift workers across three types of weekly shifts (morning = AM, afternoon = PM, and night) using a within-person design. In the second 3-week intervention period with the same participants and shift types, we analyzed the impact of adding a daily serving of walnuts to their habitual diet.

### 2.1. Participants

We included shift workers with weekly rotations (usually Monday to Friday) alternating in any order between (1) early morning, (2) afternoon, and (3) night. Shift duration ranged between 8 and 9 h. For most participants, morning shifts typically started between 04:00 and 05:00 and ended between 12:00 and 14:00; afternoon shifts started between 12:00 and 14:00 and ended between 20:00 and 22:00; and night shifts started between 20:00 and 22:00 and ended between 04:00 and 06:00. Participants were aged 18 to 65 years and had worked shifts for at least three months prior to enrollment. We recruited a convenience sample from companies employing shift workers in the French-speaking part of Switzerland, specifically in the food, pharmaceutical, machinery, and energy industries. Participants were excluded if they had used antibiotics or immunomodulators within the previous three months or during the study, were taking prebiotic or probiotic supplements, had a diagnosis of inflammatory bowel disease, had undergone major gastrointestinal surgery, or had a nut allergy. Given the marked inter-individual variability in GM composition [20], each participant served as their own control. Therefore, we did not restrict recruitment based on other potential determinants such as age or body size. Data collection began in May 2021 and ended in February 2022. Due to the COVID-19 pandemic, some participants experienced unexpected changes in their work schedules. For these individuals, data collection was divided into several weekly periods instead of the planned six consecutive weeks, extending the total participation from six to 18 weeks. In addition, some participants had 4-day instead of 5-day shifts. Among the 15 male shift workers enrolled in the study, one participant who completed only the observational phase and another with an atypical work schedule were excluded from the analysis.

### 2.2. Study Procedures

After verification of eligibility criteria and signed informed consent, a study collaborator visited each participant to provide study material and measure weight and height. During the observational phase (weeks 1, 2, and 3), participants collected stool samples at the beginning (typically Monday) and end (typically Friday) of each shift (AM, PM, Night). They used the provided Feces Catcher (Zymo Research, Irvine, CA, USA) and dedicated kits (OMNIgene·GUT, DNA Genotek, Inc., Ottawa, ON, Canada). Samples were mailed within 24 h to the research team, stored in a refrigerator, and sent to the laboratory in weekly batches. In addition, participants photographed all foods and beverages consumed each day (Monday to Sunday) using their smartphones.

During the intervention phase (weeks 4, 5, and 6), participants continued the same procedures and added one daily 30 g serving of plain walnuts (unsalted, unroasted) to their habitual diet (Monday to Sunday). Serving size was based on Swiss dietary guidelines [26]. Walnuts were pre-portioned and packaged in small, daily labelled bags. Participants were instructed to maintain their habitual diet throughout the entire week, including weekends.

**Figure 1:**
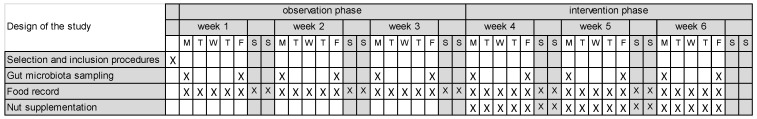
Study design and timeline across work shift. X indicates the time points at which selection and inclusion procedures, GM sampling, food record collection and walnut supplementation were performed. Shaded areas represent weekends.

Figure 1 presents the study design with the observation phase (weeks 1–3) and the intervention phrase (weeks 4–6) each including three work shifts, in a variable sequence across participants. This study was approved by the Geneva Cantonal Ethics Committee on Research Involving Humans (project ID 2020-02730, approved on 15 March 2021) and registered in the Swiss National Clinical Trial Portal (SNCTP000004312, 15 March 2021) and ClinicalTrials.gov (NCT04918537, 12 April 2021).

### 2.3. Gut Microbiota Analysis

Once received, stool samples were frozen at −80 °C and stored until processing. DNA extraction was carried out from 250 µL stool suspensions (150 samples) along with three negative controls (OMNIgene·GUT buffer) using the ZymoBIOMICS DNA Miniprep kit (Zymo Research) with a 20 min bead-beating step. Purified DNA was quantified using the Qubit dsDNA BR Assay Kit (Thermo Fisher Scientific, Carlsbad, CA, USA) and stored at −20 °C.

For PCR amplification of the bacterial 16S rRNA gene V3–4 region, 3 ng of extracted DNA (or 1 µL of the negative control extract) was used, as described previously [27]. Subsequently, MiSeq (Illumina, San Diego, CA, USA) 2 × 300 amplicon sequencing was performed following the procedures described by Lazarevic et al. [28].

Sequencing reads (ENA accession PRJEB98629) merged by PEAR [29] (-m 470 -n 390 -v50 –u 0 -p 0.0001) with average Q-score ≥ 30, as assessed by Mothur [30], were clustered into zero-radius operational taxonomic units (zOTUs) using UNOISE3 from USEARCH v. 11.0.667 pipeline [31,32].

To obtain a filtered dataset, we removed zOTUs matching any of the following criteria: (1) presented <90% identity to the reference EzBioCloud 16S database [33] sequences as revealed by USEARCH [34] (-id 0.90 -query_cov 0.99) and (2) had a <0.01% average abundance calculated for all samples. zOTUs were classified using EzBioCloud 16S database via Mothur (method = wang cutoff = 80). Decontamination of the dataset was not required as the two major contaminants, unclassified *Cutibacterium* and *Microbacterium* zOTUs, were identified under the 0.01% average abundance threshold and as such were not included in the analysis.

### 2.4. Dietary Intake and Other Variables

Because GM composition is closely associated with diet, participants’ food intake was analyzed from Monday to Friday throughout the six weeks of the study (30 days). Based on photographs submitted by the participants, an experienced dietitian recorded the date, time, type, and estimated quantity of foods and beverages consumed. Using the validated electronic food record “e-CA” [35], the dietitian manually entered the data from the pictures into the software while verifying incomplete information (e.g., type of dressing on salads and addition of oils and fats). From these records, we calculated (1) food group intake (in servings) following the Swiss dietary guidelines [26] and (2) energy as well as macro- and micronutrient intake using the Swiss Food Composition Database [36]. To evaluate overall dietary habits, we used the “Plan National Nutrition et Santé Guideline Score” (PNNS-GS) [37], a validated score of adherence to French dietary guidelines (largely consistent with Swiss recommendations), because there is no validated score based on Swiss dietary guidelines.

In addition, we recorded age, use of medications, smoking status, and self-reported physical activity (number of hours of light or intense physical activity during each type of shift and free time). Participants wearing light clothes (empty pockets) and no shoes were weighed on a scale (SECA 877) to the nearest 0.1 kg. Height was measured standing to the nearest 0.1 cm using a portable stadiometer (SECA 217). Body Mass Index (BMI) was calculated (body weight divided by height squared (kg/m^2^)) and was categorized according to the World Health Organization definition [38].

### 2.5. Statistical Analysis

Hypothesized GM changes in diversity and composition were assessed by comparing end-of-shift (Friday) samples with (i) samples from the start of the same shift (Monday) within the same study phase (observation/intervention) and (ii) end-of-shift (Friday) samples collected from a different shift type (AM, PM, Night) during the same study phase (observation/intervention).

Alpha diversity was examined using the Shannon index, which reflects both the taxonomic richness and evenness. This index was calculated after rarefaction to 26,400 reads per sample using the rrarefy function from the R vegan v2.7-1 package [39]. To evaluate beta-diversity, we compared bacterial community composition across samples through principal coordinates analysis (PCoA) and permutational analysis of variance (PERMANOVA). These analyses, based on Bray–Curtis similarity [40] of square-root-transformed relative abundances of zOTUs, were performed in PRIMER v7 (PRIMER-e, Auckland, New Zealand).

Differences in the relative abundance of individual taxa were tested for statistical significance using DESeq2 v1.44.0 [41], filtering out taxa found in less than 25% of the compared samples and adjusting for participants, if necessary. A Benjamini–Hochberg corrected *p*-value < 0.05 of the DESeq2 output was considered significant.

We first compared food group and nutrient intake between the types of shifts and study periods using linear mixed models with the subject ID as a random effect. The independent variables were shift type (AM, PM, Night) in interaction with the study period (observation, walnut supplementation). We further analyzed the diversity and composition of GM depending on the intake of food groups and nutrients known to influence GM. Spearman’s correlation was used to assess the association between overall diet (PNNS-GS) and GM diversity (Shannon index). Finally, using median values as a cut-off, we compared GM diversity (Shannon index) of participants with high or low consumption of fruit and vegetables, nuts, regular and whole grains, sugar-sweetened beverages, and meat, as well as fiber, total fats, alcohol, and PNNS-GS using Wilcoxon rank sum tests.

## 3. Results

### 3.1. Participant Characteristics

Our final sample included 13 participants with a mean (±SD) age of 42.6 ± 11.1 years. The mean BMI was 27.1 ± 5.5 kg/m^2^. Four participants had a BMI within the normal range, seven within the overweight range, and two within the obesity range. Participants had been working shifts for a mean duration of 7.6 ± 7.2 years, ranging from one to 25 years. Detailed participant-level information is provided in Supplementary Table S1.

### 3.2. Bacterial Diversity of the Gut Microbiota

The filtered 16S rRNA amplicon sequence dataset was represented by 9,548,802 reads with a median of 63,092 reads per stool sample (range 26,484–104,197). Reads were clustered into 676 operational zOTUs belonging to 13 phyla.

**Figure 2:**
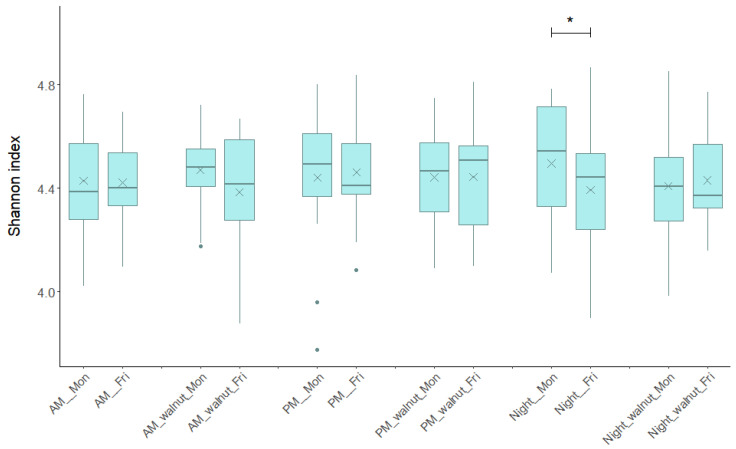
Bacterial diversity measured as Shannon diversity index based on operational taxonomic units (zOTUs) across each type of shift (AM, PM, Night), beginning/end (Monday/Friday) of the shift and for the observational/walnut-intervention phase. AM—morning shift; PM—afternoon shift; Night—night shift; Mon—Monday; Fri—Friday; walnut—intervention period. In box plots, the horizontal line represents the median and the × symbol the mean. An asterisk indicates statistically significant changes (Wilcoxon signed-rank test *p* < 0.05).

Bacterial diversity measured as the Shannon diversity index remained consistent over the study period, regardless of the shift type, day of the week (Monday or Friday), or study phase (observation, walnut supplementation) (Figure 2). The median values for the 12 groups of samples ranged from 4.371 to 4.542. No statistically significant differences were observed in the within-individual comparisons between Friday and Monday for the same shift and study phase, except during the night shift in the observation phase (no walnuts). During this shift, bacterial diversity showed a decreasing trend (*p* = 0.03, Wilcoxon signed-rank test), whereas the night shift with walnut supplementation did not show significant variation. No significant differences were found when comparing Friday samples between AM, PM and Night shifts under the same intervention regimen, indicating no change in GM diversity by shift type.

**Figure 3:**
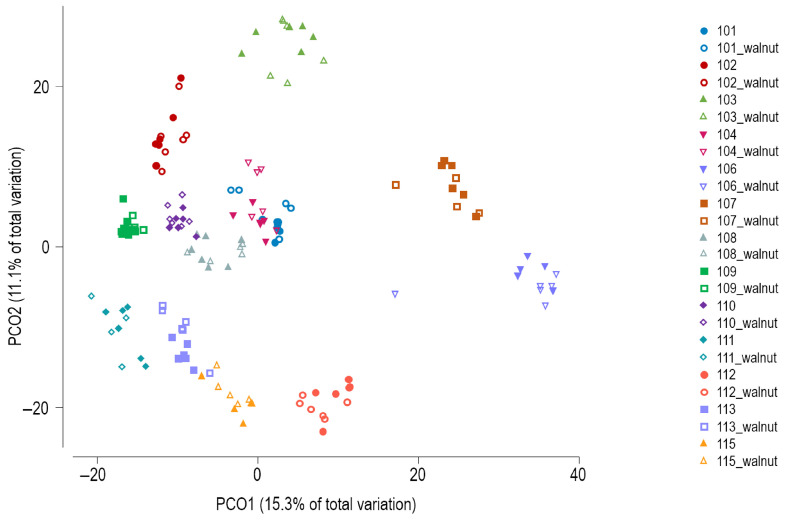
Principal coordinates analysis of Bray–Curtis similarity of bacterial communities (zOTU level). Walnut—intervention period.

Principal coordinates analysis (PCoA) of Bray–Curtis similarity showed subject-specific clustering of the GM profiles (Figure 3), which was confirmed by PERMANOVA (global test, *p* = 0.0001). PERMANOVA results indicated that none of the three factors (beginning vs. end of shift, AM/PM/night shift type, or observation vs. walnut intervention) produced significant differences in the overall microbial community composition when tested separately within specific levels of the other two factors.

### 3.3. Composition of the Gut Microbiota

**Figure 4:**
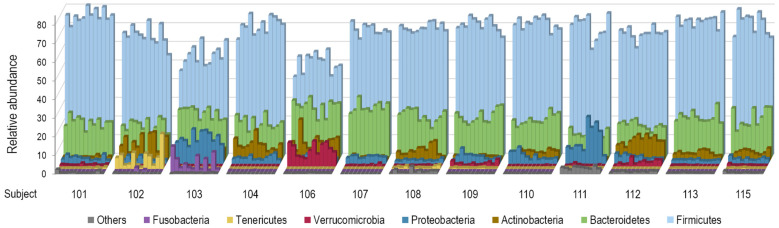
Variations of taxonomic profiles at the phylum level among the 13 study participants. Stool samples are grouped by participant in chronological order. The category “Other” comprises the following phyla: Synergistetes (syn. Synergistota), Elusimicrobia (syn. Elusimicrobiota), Spirochaetes (syn. Spirochaetota), Saccharibacteria_TM7 (syn. Candidatus Saccharimonadota), Cyanobacteria (syn. Cyanobacteriota), and Lentisphaerae (syn. Lentisphaerota).

The dominant phyla Firmicutes (syn. Bacillota [42]), Bacteroidetes (syn. Bacteroidota), Proteobacteria (now corresponding to Pseudomonadota, Bdellovibrionota, Thermodesulfobacteriota, Campylobacterota, and Myxococcota), and Actinobacteria (syn. Actinomycetota) together represented 81.2–99.9% of sequence reads in all samples. Tenericutes (syn. Mycoplasmatota), Verrucomicrobia (syn. Verrucomicrobiota), and Fusobacteria (syn. Fusobacteriota) were absent from some samples or individuals. Figure 4 shows marked inter-individual and relatively low intra-individual variations of taxonomic profiles at the phylum level among the participants.

Although global analyses of microbiota structure and complexity revealed no significant differences by beginning vs. end of the shift, shift type, or walnut consumption, several bacterial taxa were differentially abundant across these conditions. The phylum Lentisphaerae, including its associated lower-level taxa, and genera from the order Clostridiales (phylum Firmicutes), notably *Oscillibacter*, PAC001609_g, and Eubacterium_g23, were more abundant at the end of work shifts (Friday) when shifts later in the day were compared to earlier shifts (PM vs. AM, Night vs. AM, Night vs. PM) (Supplementary Figure S1). In contrast, the abundance of the family Sutterellaceae was significantly decreased in these comparisons. A similar pattern was observed in Friday-to-Monday comparisons within shifts, with Lentisphaerae, *Oscillibacter*, and Eubacterium_g23 increasing toward the end of the week, while Sutterellaceae decreased over the same period (Supplementary Figure S2).

### 3.4. Food Intake

Based on 30-day food records, participants consumed a mean total energy intake of 2413 ± 452 kcal/day, with no significant differences across shift types. The mean healthy eating score (PNNS-GS score) was 7.2 ± 1.2 (range 5.3–9.0). The mean fiber intake was 21.8 ± 5.6 g/day, below the recommended 30 g/day [26]. As expected, walnut and lipid intake was higher during the three weeks of intervention, but fiber intake did not differ significantly between the observational and intervention phases. During the intervention phase of walnut supplementation, daily energy intake increased by 152 ± 73 kcal compared to the observational phase (*p* = 0.04, Table 1).

**Figure 5:**
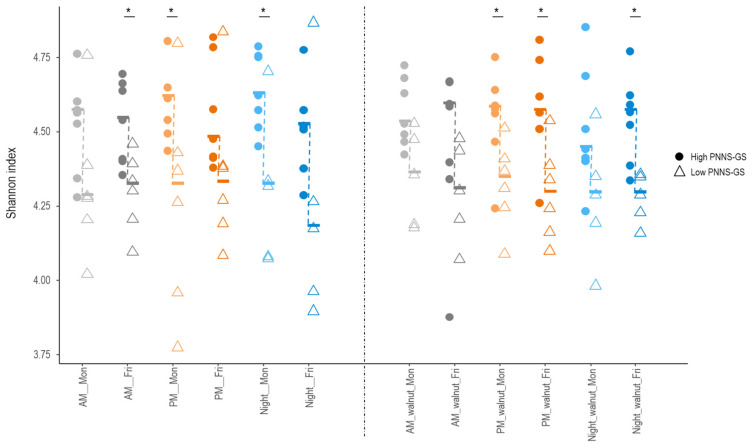
Differences in GM diversity across 12 study conditions in participants following nutritional recommendations more (High PNNS-GS) or less (Low PNNS-GS) closely. Median values (symbol —) are connected by a dashed line. AM—morning shift; PM—afternoon shift; Night—night shift; Mon—Monday; Fri—Friday; walnut—intervention period; PNNS-GS—Plan National Nutrition et Santé Guideline Score. An asterisk indicates statistically significant differences between participants with low vs. high PNNS-GS (Wilcoxon rank sum test *p* < 0.05).

Across the entire study period, adherence to dietary guidelines (PNNS-GS score) was positively correlated with GM alpha diversity, as measured by the Shannon index (r = 0.64, *p* = 0.02). Indeed, diversity was higher in participants with a higher PNNS-GS score, compared to those with a lower score across all 12 conditions studied, with significant differences (Wilcoxon rank sum test, *p* < 0.05) in six of them (Figure 5). In contrast, the Shannon index did not differ significantly according to fiber, total fat, alcohol intake, or any of the tested food groups. Several bacterial taxa differed in abundance between study participants with high and low PNNS-GS scores (Supplementary Figure S3). When differences in a given taxon were observed at multiple time points, they consistently followed the same direction.

## 4. Discussion

In this study, we compared GM diversity and composition across different shift types and evaluated the effects of walnut supplementation in shift workers. We found no significant overall changes in GM diversity or composition associated with shift type or walnut supplementation. However, a decrease in diversity was observed at the end of the night shift during the observational phase, which was not observed after walnut supplementation. Across the study, adherence to a healthier diet was positively associated with greater GM diversity.

Our findings are consistent with the limited literature on shift work and GM, which has generally reported minimal effects on overall microbial diversity. Mortas et al. [43], who followed 10 security workers and analyzed GM composition after four weeks of day work (07:00–15:00) or two weeks of night shift (23:00–07:00), found no significant changes in alpha or beta diversity. Interestingly, they observed that some participants showed stronger individual responses to circadian disruptions induced by shift work, although no significant group-level differences were detected. Similarly, Benedict et al. (2016) studied nine healthy males exposed to two nights of partial sleep deprivation (PSD), a common feature of shift work, and found no significant difference in beta diversity [44]. In our study, we found that alpha diversity was significantly higher in participants who adhered more closely to healthy eating guidelines, what is consistent with the results of a long-term multi-ethnic cohort, in which increased alpha-diversity was significantly associated with a healthier diet, even in the long term [45].

Regarding GM composition, Mortas et al. reported a decrease in the relative abundance of Bacteroidetes and an increase in Actinobacteria and Firmicutes levels after night shift [43]. In their study, *Faecalibacterium*, known to play a protective role against gut inflammation and reduce bacterial translocation, emerged as a biomarker of day shift, whereas *Dorea longicatena* and *Dorea formicigenerans* were more abundant during night shifts. Benedict et al. observed that PSD increased the Firmicutes:Bacteroidetes ratio, increased the relative abundance of the families Coriobacteriaceae and Erysipelotrichaceae, and decreased the relative abundance of Tenericutes [44]. In our study, several taxa, notably Lentisphaerae, *Oscillibacter*, Eubacterium_g23, and Sutterellaceae appeared as potential markers of work shifts. Interestingly, the progression within each shift week (Monday to Friday) mirrored some of the differences between shift types (AM, PM, Night), suggesting a shared underlying influence on microbial composition, which could hypothetically include factors such as accumulated fatigue.

Interpreting changes in GM composition remains challenging because distinguishing healthy from unhealthy profiles is not straightforward. In fact, the concept of “healthy microbiome” is debated, and some experts advocate focusing instead on the functions the microbiota performs for the host [46]. Moreover, the “ideal” microbiome is likely to evolve dynamically depending on factors such as age, dietary habits, or physiological state [46]. Beyond composition, GM rhythmicity itself may be an independent risk factor, as shown for type 2 diabetes [47]. This highlights the importance of considering both the functional and temporal aspects of the GM when studying populations exposed to circadian disruption, such as shift workers.

Despite the uncertainty about how to define a healthy microbiome, diet remains a central modulator shaping GM composition and diversity. Undigested dietary components such as fiber, fats, proteins, and complex carbohydrates serve as substrates for specific bacterial groups. High-fiber diets, primarily derived from fruits, vegetables, and whole grains, are associated with increased microbial diversity [48]. Fibers serve as prebiotics, promoting the growth of beneficial bacteria that contribute to the overall gut health. Many interventional studies to date have used fiber supplements, such as fructooligosaccharides or galactooligosaccharides, rather than whole food. However, plant-based whole foods, such as nuts, contain, in addition to fiber, a wide range of vitamins, minerals, and phytochemicals (e.g., flavonoids, isoflavonoids, lignins, stilbenoids, tannins, and polyphenols) that may also influence GM composition [48]. Growing evidence indicates that nut consumption can confer beneficial effects on oxidative stress, inflammation, and vascular reactivity [49]. A meta-analysis of nine randomized clinical trials reported that nut consumption increased the abundance of *Clostridium*, *Dialister*, *Lachnospira*, and *Roseburia*, while decreasing *Parabacteroides* [24]. In our study, several taxa showed significant changes during work shifts in the walnut supplementation and observation phases; when the same taxa changed in both phases, the direction of the change was consistent. For example, three taxa (*Oscillibacter*, *Alistipes senegalensis*, and *Victivallis* PAC001213_s) varied significantly in two work shifts with walnut supplementation, but also in one or two shifts without supplementation. Likewise, the abundance of the phylum Lentisphaerae and class Lentisphaeria increased during the two walnut supplementation shifts, with a similar, although non-significant, trend during the observation phase. Furthermore, none of the taxa showed significant changes across all three work shifts within the same intervention type (Supplementary Figure S2), suggesting potential biological trends but with variable consistency. In our study, walnut supplementation was associated with a significant increase in total energy intake and proportion of lipids in the diet, which could raise concerns about potential for weight gain. This concern is not supported by the current literature, with two systematic reviews concluding that nut consumption was not associated with increased adiposity [50,51]. However, future studies should monitor weight dynamics during interventions based on nut supplementation.

GM composition has been shown to be bidirectionally associated with obesity, with individuals with obesity exhibiting distinct microbial profiles [52], and experimental studies demonstrating that alterations in gut microbiota can contribute to weight gain [53]. In the present study, participants displayed a wide range of BMI values (19.3–34.2 kg/m^2^), which may partly account for the observed interindividual variability in GM composition. However, as the study employed a within-subject design in which each participant served as their own control, BMI is unlikely to have influenced the comparisons across shift types or the observed effects of walnut supplementation.

We observed a modest but significant decrease in bacterial diversity on the last day of the night shift during the observational phase but not under nut supplementation. This is interesting because higher bacterial diversity is generally associated with healthier eating patterns, and maintaining such habits is particularly challenging for night workers. Shift workers face multiple nutritional barriers, including irregular eating routines, sleep debt, circadian disruption, and reduced access to healthy foods. These factors interact to increase hunger, reduce satiety, and alter food preferences in environments where healthy food options are limited [54]. In this context, supporting shift workers to improve their diet quality should be a central goal in reducing the risk of chronic diseases. Strategies include promoting a diet rich in fiber and whole foods and modulating the timing of food intake as an additional preventive approach.

This study has several methodological and practical strengths. The longitudinal design with repeated sampling per participant enabled monitoring of within-person changes over time. By focusing on a shift pattern commonly encountered across industries and using whole-food intervention, the study enhances the real-world relevance of the findings. On a methodological level, the use of multiple diversity metrics, as recommended [55] increases robustness. Finally, the comprehensive assessment of dietary intake throughout the study period allowed for a detailed characterization of food consumption patterns.

Several limitations should however also be acknowledged. The small sample size, largely due to recruitment challenges in the context of the COVID-19 pandemic, may have limited the statistical power to detect subtle effects, including those related to walnut supplementation. Although the within-person design helped control for some variability, non-consecutive study weeks and heterogeneity in shift schedules may have introduced additional variability. Moreover, seasonal influences on the gut microbiota composition cannot be ruled out [56].

## 5. Conclusions

In this study, we found no consistent effects of morning, afternoon, or night shifts on the overall GM diversity or composition. Likewise, walnut supplementation had no major impact, although it appeared to preserve microbial diversity at the end of night shifts, when a decline was otherwise observed during the observational phase.

Several taxa showed differential abundances across shifts and study phases, suggesting that at least some changes may reflect real biological trends rather than random variation. Importantly, adherence to healthier dietary patterns was consistently associated with greater microbial diversity.

Future studies should explore interventions that target both dietary quality and meal timing using larger and more diverse samples to further elucidate the complex bidirectional relationships between diet, circadian rhythms, and gut microbiota.

## Figures and Tables

**Table 1:** Energy, nutrient and food group intake (means ± SD of five days food records) and gut microbiota diversity (Shannon index) by type of shift and for the observation and intervention periods (N = 13). AM—morning, PM—afternoon, SD—standard deviation, TEI—total energy intake, serv—serving, SSB—sugar-sweetened beverages, PNNS-GS—Plan National Nutrition et Santé Guideline Score, Mon—Monday, Fri—Friday.

	Observation (Obs)			Intervention (Interv)			Obs vs. Interv	AM vs. PM vs. Night	Within-Subject	Between-Subject
	AM	PM	Night	AM	PM	Night	*p*	*p*	SD	SD
Energy (kcal)	2267 ± 557	2280 ± 355	2463 ± 489	2407 ± 560	2473 ± 603	2570 ± 695	0.04	0.09	320	420
Proteins (% TEI)	15.5 ± 2.8	16.2 ± 2.3	16.2 ± 3.4	15.6 ± 3.5	16.0 ± 3.1	16.0 ± 2.7	0.80	0.63	2.1	2.0
Lipids (%TEI)	39.0 ± 5.7	40.5 ± 3.3	39.4 ± 3.9	45.9 ± 8.4	44.2 ± 4.7	45.1 ± 4.2	0.00	0.97	4.4	2.6
Carbohydrates (% TEI)	42.1 ± 6.0	40.1 ± 4.5	41.3 ± 5.1	35.0 ± 7.4	37.2 ± 6.3	35.6 ± 5.8	0.00	0.99	4.3	3.8
Fiber (g)	20.8 ± 8.6	21.8 ± 5.3	21.9 ± 5.9	20.7 ± 6.5	23.9 ± 8.2	22.0 ± 5.0	0.53	0.14	3.9	5.2
Alcohol (g)	5.8 ± 8.7	4.6 ± 6.4	6.6 ± 10.1	7.3 ± 9.8	2.4 ± 4.9	7.2 ± 11.8	0.89	0.01	4.4	7.3
Nuts (serv)	0.37 ± 0.39	0.23 ± 0.38	0.33 ± 0.31	1.49 ± 0.32	1.67 ± 0.47	1.65 ± 0.40	0.00	0.75	0.30	0.22
Fruit-vegetables (serv)	2.88 ± 1.32	3.58 ± 2.04	3.43 ± 1.62	2.37 ± 1.45	2.66 ± 0.96	2.73 ± 1.54	0.00	0.04	0.74	1.27
Whole grains (serv)	0.18 ± 0.36	0.27 ± 0.24	0.28 ± 0.34	0.15 ± 0.14	0.60 ± 1.75	0.21 ± 0.30	0.59	0.32	0.67	0.33
SSB (mL)	184 ± 308	169 ± 315	230 ± 441	155 ± 307	138 ± 317	135 ± 220	0.30	0.05	105	315
PNNS-GS score	7.6 ± 1.3	7.2 ± 1.7	7.6 ± 1.6	7.9 ± 1.2	8.1 ± 1.3	7.5 ± 1.1	0.17	0.89	1.2	0.6
Shannon index (Mon)	4.43 ± 0.22	4.44 ± 0.30	4.50 ± 0.25	4.47 ± 0.17	4.44 ± 0.19	4.41 ± 0.23	0.73	0.81	0.13	0.19
Shannon index (Fri)	4.42 ± 0.18	4.46 ± 0.23	4.39 ± 0.29	4.38 ± 0.24	4.44 ± 0.22	4.43 ± 0.18	0.74	0.28	0.13	0.18

## Data Availability

The original data presented in the study will be openly available in Yareta at https://doi.org/10.26037/yareta:moawrjzm45hdhnokrtefy77u5i and European Nucleotide Archive (ENA; www.ebi.ac.uk/ena) under the study number PRJEB98629.

## Supplementary Materials

The following supporting information can be downloaded at: https://www.mdpi.com/article/10.3390/ijerph23020169/s1, Supplementary Table S1: Detailed individual characteristics of participants; Supplementary Figure S1: Comparison of gut microbiota composition between shifts; Supplementary Figure S2: Comparison of gut microbiota composition between the beginning and end of each shift; Supplementary Figure S3: Comparison of gut microbiota composition between participants with a healthier diet (high PNNS) and a less healthy diet (low PNNS).

## Abbreviations

The following abbreviations are used in this manuscript:

AM	Morning shift
BMI	Body mass index
Fri	Friday (end of shift)
GM	Gut microbiota
Mon	Monday (beginning of shift)
PM	Afternoon shift
PNNS-GS	Plan National Nutrition et Santé Guideline Score
PSD	Partial sleep deprivation
zOTU	zero-radius operational taxonomic units
