# Supplementary material for: Gut Microbiota Diversity and Composition Across Shift Types and the Effects of Walnut Supplementation—An Observational and Interventional Study
Source: Int J Environ Res Public Health. 2026 Jan 29;23(2):169. doi: 10.3390/ijerph23020169 (PMC12940553; doi:10.3390/ijerph23020169)
Supplement: Supplementary file 1 [file ijerph-23-00169-s001.zip › ijerph-4041163-supplementary.pdf]

Supplementary Table S1 : Detailed individual characteristics of participants.

Subject		101	102	103	104	106	107	108	109	110	111	112	113	115
Age (years)		46	58	38	29	60	32	46	51	41	54	32	52	28
BMI (kg/m^2)		29.3	27.4	26.8	23.6	26.3	39.6	27.7	20.4	29.4	34.2	22.4	25.4	19.3
Duration prior shift work (years)		18	7	4	8	7	1	1	26	16	8	6	7	3
Work shift schedule – observation	W1 (I)<br>W2 (I)<br>W3 (I)	N (0)<br>PM (0)<br>AM (0)	N (0)<br>PM (0)<br>AM (0)	PM (0)<br>N (0)<br>AM (4)	PM (0)<br>AM (0)<br>N (9)	N (0)<br>AM (0)<br>PM (5)	PM (0)<br>N (0)<br>AM (1)	PM (0)<br>N (0)<br>AM (0)	N (0)<br>AM (0)<br>PM (12)	N (0)<br>PM (0)<br>AM (1)	N (0)<br>PM (0)<br>AM (0)	N (0)<br>PM (0)<br>AM (3)	N (0)<br>PM (0)<br>AM (3)	AM (0)<br>PM (0)<br>N (6)
Work shift schedule – intervention	W4 (I)<br>W5 (I)<br>W6 (I)	PM (0)<br>N (0)<br>AM	PM (0)<br>N (3)<br>AM	AM (0)<br>PM (0)<br>N	PM (0)<br>N (0)<br>AM	N (0)<br>AM (0)<br>PM	AM (0)<br>PM (0)<br>N	PM (0)<br>N (0)<br>AM	AM (0)<br>N (0)<br>PM	AM (0)<br>N (0)<br>PM	N (1)<br>PM (0)<br>AM	N (0)<br>AM (0)<br>PM	N (0)<br>AM (0)<br>PM	PM (0)<br>AM (2)<br>N

BMI, Body Mass Index; W, Week; I, Interval with the following studied shifts (in weeks); N, Night shift; AM, morning shift; PM, afternoon shift.

Supplementary Figure S1: Comparison of gut microbiota composition between shifts.

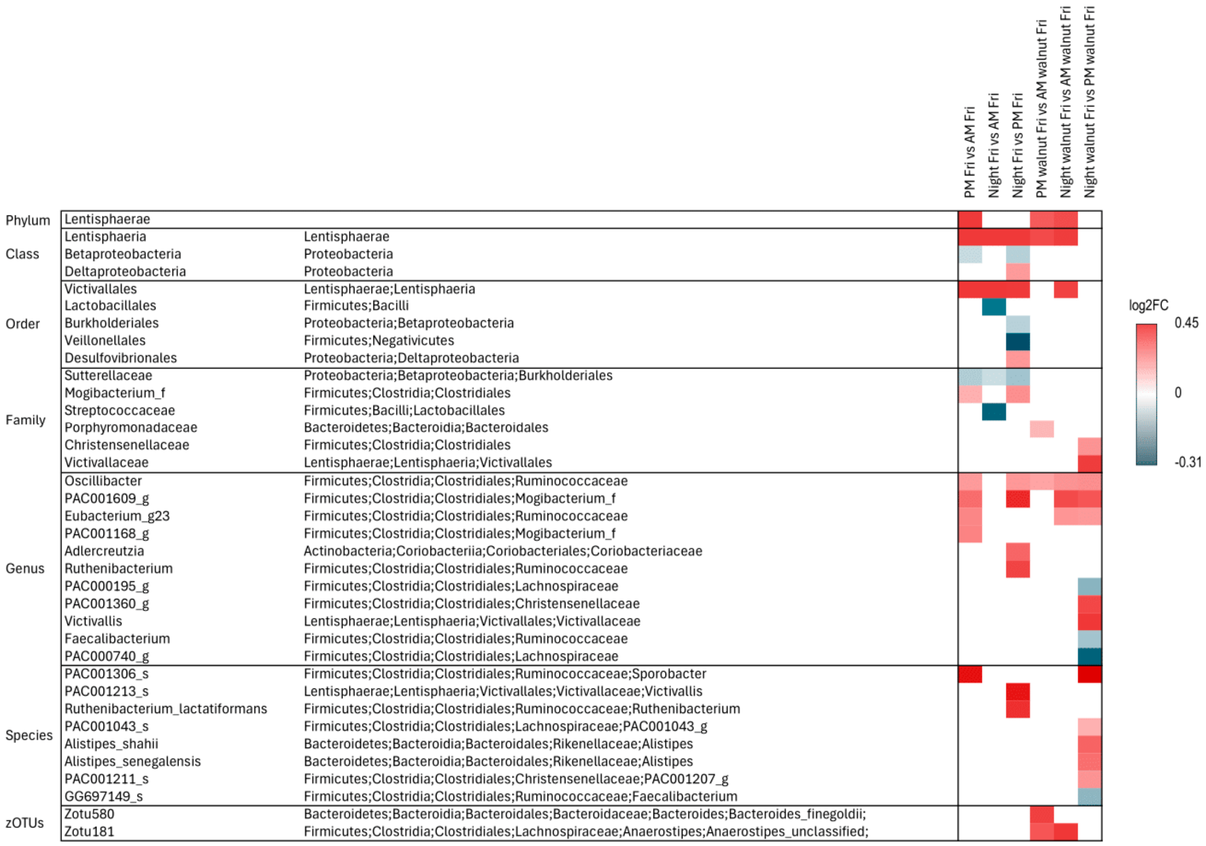

Supplementary Figure S1: Differences in relative abundances of bacterial taxa between Fridays of each type of shift (AM, PM, Night) and intervention (walnut, observation), assessed by DESeq. Only statistically significant changes are presented. The heat map colors indicate these changes, with blue representing a decrease and red representing an increase in the first named type of shift relative to the second named type of shift. Color intensity corresponds to the fold change. AM, morning shift; PM, afternoon shift; Night, night shift; Fri, Friday; walnut, intervention period.

Supplementary Figure S2: Comparison of gut microbiota composition between the beginning and end of each shift.

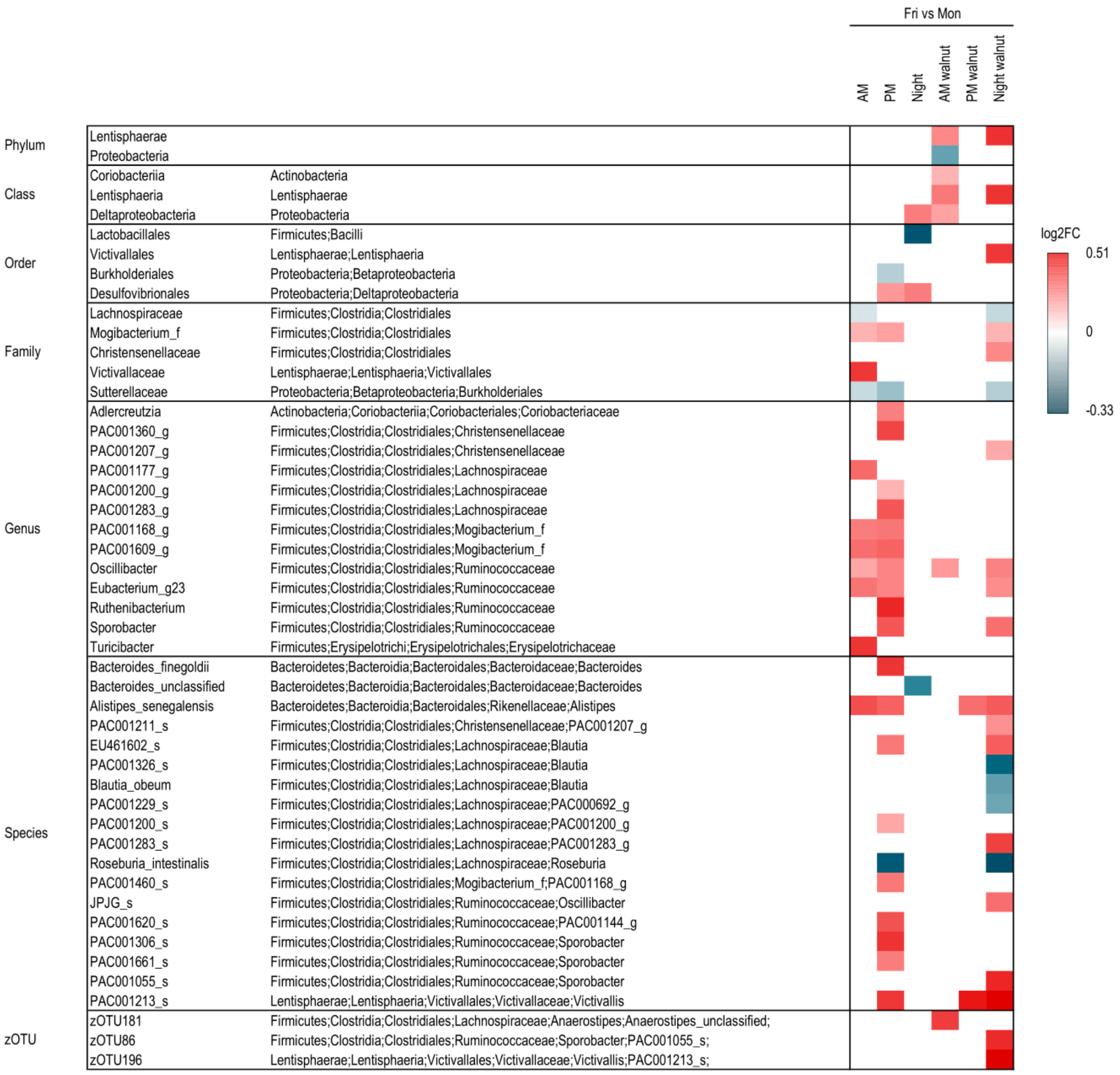

Supplementary Figure S2: Differences in relative abundances of bacterial taxa between the beginning (Monday) and end (Friday) of each shift (AM, PM, Night) and intervention type (observation, walnut supplementation), assessed by DESeq. Only statistically significant changes are presented. The heat map colors indicate these changes, with blue representing a decrease and red representing an increase in the end of the shift (Friday) relative to the beginning (Monday). Color intensity corresponds to fold change. AM, morning shift; PM, afternoon shift; Night, night shift; Mon, Monday; Fri, Friday; walnut, intervention period.

Supplementary Figure S3: Comparison of gut microbiota composition between participants with a healthier diet (high PNNS-GS) and a less healthy diet (low PNNS-GS).

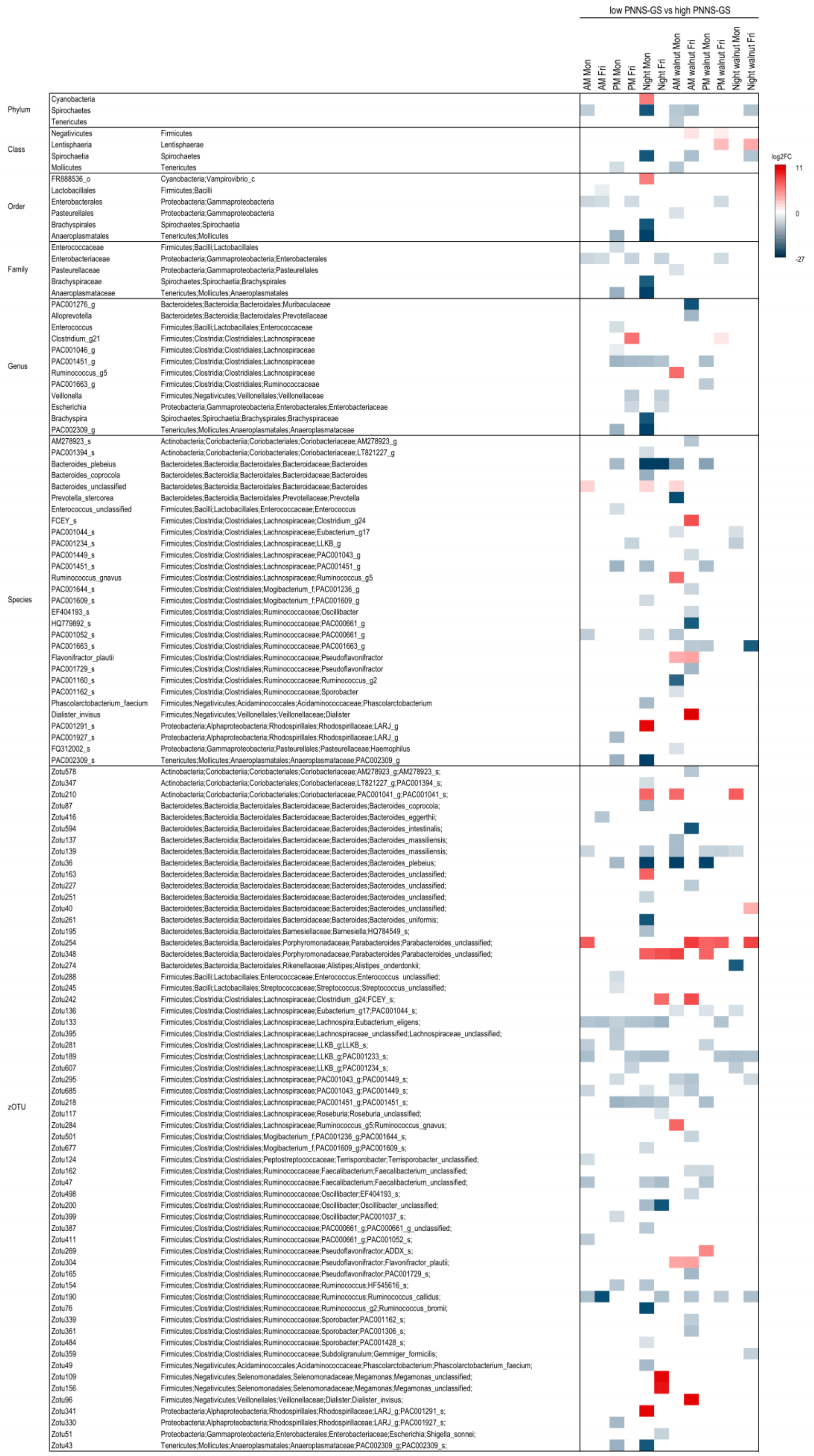

Supplementary Figure S3: Differences in bacterial diversity and relative abundances of bacterial taxa between low and high PNNS-GS participants, assessed by DESeq. Only statistically significant changes are presented. The color heat map indicates these changes, with blue representing a decrease and red representing an increase in low PNNS-GS relative to high PNNS-GS. Color intensity corresponds to the fold change (scale on the right). AM, morning shift; PM, afternoon shift; Night, night shift; Mon, Monday; Fri, Friday; walnut, intervention period; PNNS-GS, Plan National Nutrition et Santé Guideline Score.
